# Observations of greenhouse gases as climate indicators

**DOI:** 10.1007/s10584-021-03001-7

**Published:** 2021-03-08

**Authors:** Lori Bruhwiler, Sourish Basu, James H. Butler, Abhishek Chatterjee, Ed Dlugokencky, Melissa A. Kenney, Allison McComiskey, Stephen A. Montzka, Diane Stanitski

**Affiliations:** 1 NOAA Global Monitoring Laboratory, Boulder, CO USA; 2 NASA Goddard Space Flight Center, Greenbelt, MD USA; 3 Universities Space Research Association, Columbia, MD USA; 4 University of Minnesota Institute on the Environment, Saint Paul, MN USA; 5 Brookhaven National Laboratory, Environmental & Climate Sciences Department, Upton, NY USA

**Keywords:** Greenhouse gases, Radiative forcing, Anthropogenic emissions, Atmospheric composition

## Abstract

Humans have significantly altered the energy balance of the Earth’s climate system mainly not only by extracting and burning fossil fuels but also by altering the biosphere and using halocarbons. The 3rd US National Climate Assessment pointed to a need for a system of indicators of climate and global change based on long-term data that could be used to support assessments and this led to the development of the National Climate Indicators System (NCIS). Here we identify a representative set of key atmospheric indicators of changes in atmospheric radiative forcing due to greenhouse gases (GHGs), and we evaluate atmospheric composition measurements, including non-CO_2_ GHGs for use as climate change indicators in support of the US National Climate Assessment. GHG abundances and their changes over time can provide valuable information on the success of climate mitigation policies, as well as insights into possible carbon-climate feedback processes that may ultimately affect the success of those policies. To ensure that reliable information for assessing GHG emission changes can be provided on policy-relevant scales, expanded observational efforts are needed. Furthermore, the ability to detect trends resulting from changing emissions requires a commitment to supporting long-term observations. Long-term measurements of greenhouse gases, aerosols, and clouds and related climate indicators used with a dimming/brightening index could provide a foundation for quantifying forcing and its attribution and reducing error in existing indicators that do not account for complicated cloud processes.

## Introduction

Humans have significantly perturbed the energy balance of the climate system mainly not only by extracting and burning fossil fuels but also through land use changes and halocarbon usage. Agriculture and waste treatment also release nitrous oxide and methane to the atmosphere, and both are strong greenhouse gases (GHGs). In response to anthropogenic GHG emissions, the global atmosphere has warmed over the last half century. By 2017, global mean surface temperature (GMST) was found to be about 1 °C above preindustrial values and increasing at 0.2 °C/decade (Allen et al. 2018). This trend is slightly higher (4–8%) if global mean surface air temperature (GSAT) rather than blended air and sea surface temperature is considered (Cowtan et al. 2015; Richardson et al. 2018).

Understanding changes in atmospheric composition is essential for estimating changes in the energy balance of the atmosphere. Quantifying exchanges of GHGs among the biosphere, oceans, and atmosphere as well as anthropogenic emissions can also be useful indicators of global change but are more difficult to quantify. Time-dependent emission estimates are crucial for evaluating success of emission reduction strategies, and identifying trends in natural exchange processes can further understanding of how natural processes respond to climate change.

The 3rd US National Climate Assessment pointed to a need for a system of indicators of climate and global change based on long-term data that could be used to support assessments (Corell et al. 2014). Kenney et al. (2014, 2016) described development and prototypes of such a system, the National Climate Indicators System (NCIS), to establish a “system of physical, natural, and societal indicators that communicate and inform decisions about key aspects of the physical climate, climate impacts, vulnerabilities, and preparedness.” Its primary purpose is to support the sustained US National Climate Assessment (Buizer et al. 2013) by providing long-term information that is regularly updated and comparative to a baseline of change. Indicators are reference tools that can be constructed from measured data, modeled data, or an index and they can facilitate advancement and communication of scientific understanding, inform decision-making, and demonstrate progress in achieving management objectives. Indicators relevant to key US systems and sectors, such as indicators for the atmosphere including greenhouse gases, are required by the 1990 Global Change Research Act and are of broad concern to the US public (see Kenney et al. 2018, this issue). A proof-of-concept indicator system was released by the US Global Change Research Program in 2015 (http://www.globalchange.gov/explore/indicators) and included Annual Greenhouse Gas Index (AGGI) and Atmospheric Carbon Dioxide indicators. This paper revisits and expands on the conceptual model for these indicators.

Observations of atmospheric greenhouse gases are useful climate indicators because they can be used to show the influence of human activities (e.g., emissions) on the climate system. Carbon dioxide (CO_2_) is included in USGCRP Climate Indicators, while atmospheric burdens of methane (CH_4_), nitrous oxide (N_2_O), synthetic ozone depleting gases, and other long-lived halogenated gases and their changes over time (including over the past 800,000 years) are included in the EPA’s set of key climate indicators. Both the EPA and the USGCRP emphasize the utility of climate indicators for communication about changing climate, environmental assessment, and informed decision-making. A related effort is the collection of Essential Climate Variables (ECVs) identified by the World Meteorological Organization (WMO, https://public.wmo.int/en/programmes/global-climate-observing-system/essential-climate-variables). The criteria for ECVs include how critical a variable is for characterizing the changing climate system, and whether it is feasible and cost effective to observe that particular variable. The WMO also defines a set of climate monitoring principles that lay out needs for continuity and compatibility of data sets, data management, maintenance of long-term measurements, and focus on under-observed regions.

The objective of this paper is to identify a representative set of key atmospheric indicators of changes in atmospheric radiative forcing driven by greenhouse gases. In particular, we evaluate the use of atmospheric composition measurements as climate change indicators. We present a conceptual model of climate forcing used to identify potential indicators (Section 2), the proposed key indicators (Sections 3, 4, and 5), and research and indicator development opportunities (Section 6). Such indicators provide an overview of the climate forcing that causes the climate impacts and vulnerabilities, and, when tracked over time, can be a measure of large-scale mitigation response effectiveness.

Global surface temperature change is an essential indicator of changing climate, and climate change policies have targeted limits in global temperature increase relative to the pre-industrial era. The recognition of an approximate linear relationship between cumulative CO_2_ emissions and temperature increase has allowed for estimation of remaining CO_2_ emissions consistent with temperature change targets (Allen et al. 2009; Gregory et al. 2009; Matthews et al. 2009). Clearly, observational records from which global atmospheric burdens of CO_2_ can be deduced provide an important link between cumulative emissions, which could be considered climate indicators, and global temperature change. Such observations allow confirmation of the linear relationship (Gillett et al. 2013) and could be useful for detecting departures from the linear relationship between cumulative emissions and temperature change if feedback processes become significant.

Non-CO_2_ climate forcers or short-lived climate pollutants (SLCPs), such as CH_4_, N_2_O, and manufactured halogenated gases, complicate the simple connection between cumulative CO_2_ emissions and temperature. Some SLCPs have lifetimes as short as a decade, implying that the effects of emissions at a particular time will not significantly influence temperatures decades later. On the other hand, some short-lived climate pollutants (SLCPs) are much more effective at trapping heat in the climate system than CO_2_ so that reducing their emissions could provide short term benefit. It may also be economically advantageous to reduce SCLP emissions. For example, fugitive emissions of CH_4_ from oil and gas production have economic value. For policy considerations, it is useful to have metrics that allow tradeoffs between CO_2_ and SCLP emissions to be evaluated; however, incorporating SLCPs into cumulative carbon budgets is difficult due to the range and uncertainties of atmospheric lifetimes. Metrics designed to provide simple quantifications of the joint effects of SLCPs and CO_2_ emissions on atmospheric radiative forcing range from looking at GWPs and GTPs over short- and long-time horizons, to using simple or medium complexity models to estimate climate-relevant equivalence between SLCP and CO_2_ emissions (Tanaka et al. 2010, 2019; Jenkins et al. 2018; Cain et al. 2019; Collins et al. 2020). Observational records of the global abundances of SLCPs are essential for computing and evaluating these metrics.

## A conceptual model of climate forcing indicators

**Fig. 1:**
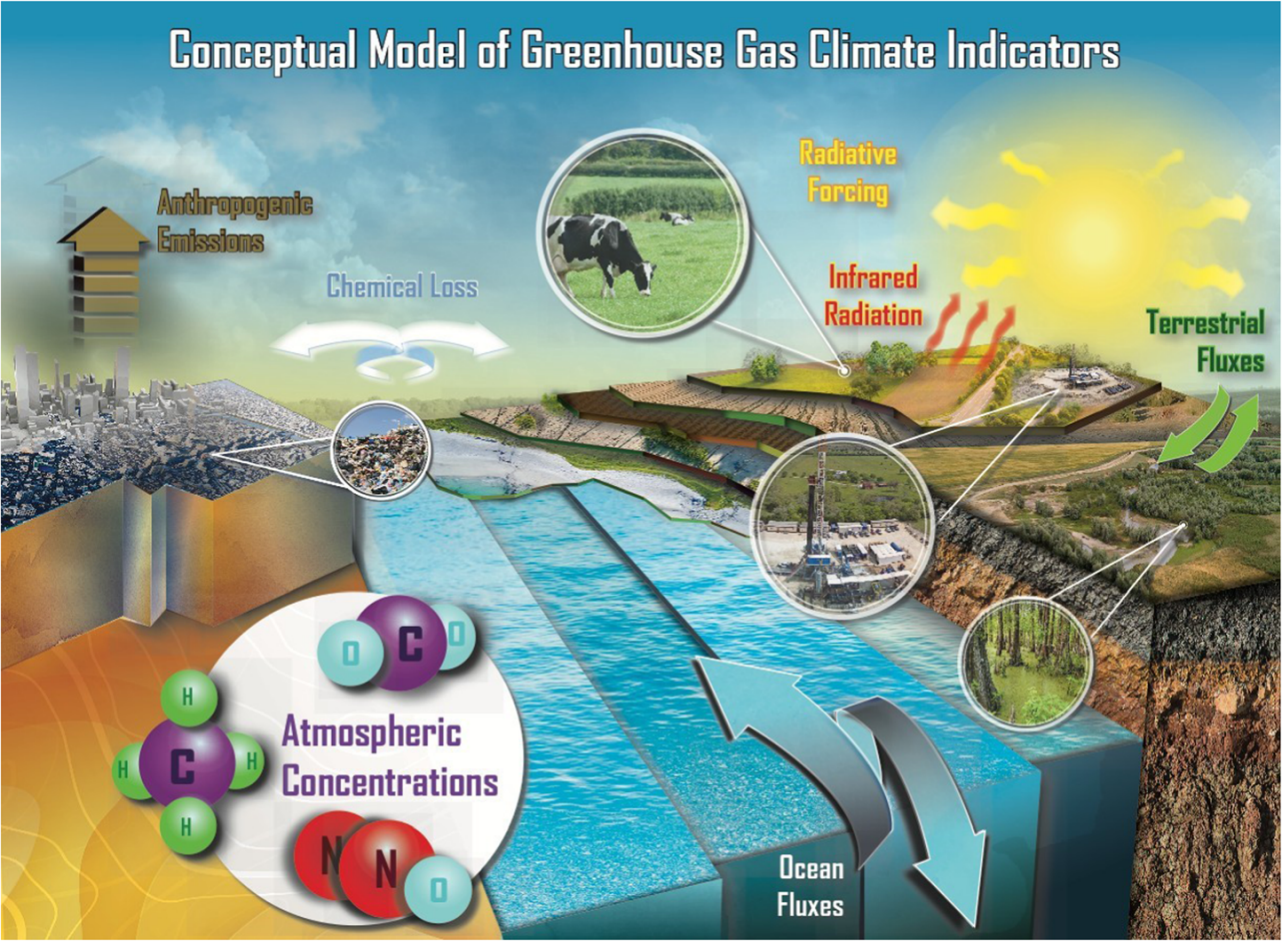
Conceptual model for greenhouse gas (GHG) climate indicators. Indicators proposed in this study are atmospheric concentrations of non-CO_2_ GHGs (CO_2_ is already included in the National Climate Indicator System (NCIS)). Atmospheric GHG concentrations are directly observable and can be monitored over time. They can also be used to estimate radiative forcing. The Annual Greenhouse Gas Index (AGGI) is also included in the NCIS and quantifies the change in radiative forcing due to GHGs relative to 1990. Solid arrows indicate sources and sinks of atmospheric GHGs. Observations constraining these processes could be useful future climate indicators, but fluxes of GHGs are at present difficult to quantify at large spatial and temporal scales

Kenney et al. (2018, this issue) point to the need for a conceptual model to justify climate indicators. A conceptual model is communicated by a simple diagram that shows how a complex system functions, and measurable quantities that can be used to characterize important components of the system. Figure 1 shows a conceptual model of atmospheric composition climate indicators. Radiative forcing is the quantity that needs to be quantified over time, and in Section 4, we describe how it can be estimated using observations of major GHGs and used as a climate indicator. We also propose that atmospheric concentrations of major non-CO_2_ GHGs are indicators of changes in anthropogenic and natural sources and sinks. Atmospheric concentrations of GHGs underlay radiative forcing estimates and change over time due to anthropogenic emissions and natural processes that regulate exchanges of these gases among atmosphere, terrestrial biosphere, and oceans (Section 3). Many SLCPs are also removed in the atmosphere by chemical or photolytic loss.

Information about fluxes, if constrained by observable quantities and updated over time, could also be useful indicators (Section 5). Additional indicators could be identified to quantify observed changes in radiative fluxes at the Earth’s surface as a foundation for understanding the role of GHGs versus the impacts of changing aerosol and cloud distributions due to changes in both emissions (aerosol) and changes in atmospheric circulations (clouds) from natural and anthropogenic processes. These future climate indicators would be more difficult to quantify and would involve the use of models, process level measurements, and inventories of emissions. As such, use of these quantities as possible future climate indicators is still an active area of research.

### Atmospheric measurements of radiatively active atmospheric constituents as climate indicators

**Fig. 2:**
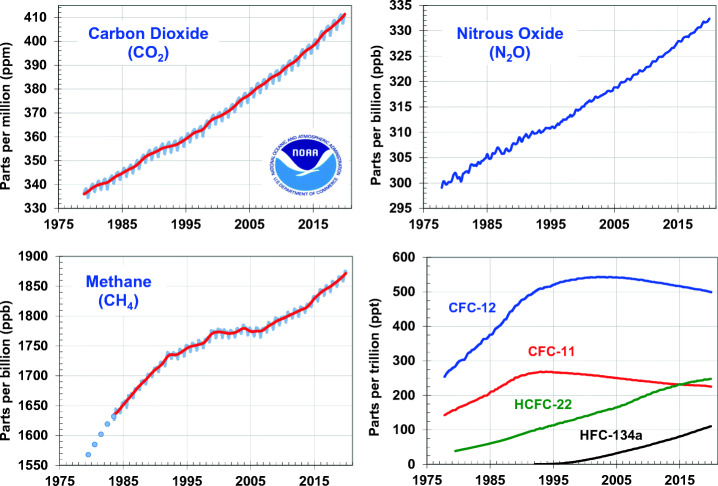
Global average abundances of the major, well-mixed, long-lived greenhouse gases—carbon dioxide, methane, nitrous oxide, CFC-12, and CFC-11—from the NOAA global air sampling network are plotted since the beginning of 1979

In this section, we review the role of major GHGs in climate forcing. We discuss the role of emission inventories for anthropogenic sources and point out their value as climate indicators. Long-term observational records of major greenhouse gases are essential for evaluating bottom-up understanding of emissions and for evaluating the effectiveness of mitigation policies (Canadell et al. 2010). Figure 2 shows observations of major greenhouse gases and how they have changed over time.

The strong greenhouse gas sulfur hexafluoride (SF_6_), a manufactured gas that is used as an electrical insulator, provides an instructive example of the importance of atmospheric measurements. Emissions of SF_6_ reported to the UNFCCC (which includes only Annex 1 Countries) were found by Rigby et al. (2010) to be much lower than those derived using global atmospheric observations of SF_6_. Monitoring atmospheric concentrations of greenhouse gases is essential as an evidence-based, independent method for assessing the effects of strategies to reduce emissions.

### Emission inventories: the “bottom-up” approach

The framework of using indicators to measure progress towards emission and sustainable development goals has long been employed by the emission inventory community. For example, the UNEP Economics and Trade Unit developed a standardized methodology for estimating GHG emissions from companies and other organizations (Thomas et al. 2000). The California Air Resources Board produces ongoing report of GHG emissions and economic indicators related to emissions (CARB 2020). Indicators in support of the UN’s sustainable development goals were described by Schmidt-Traub et al. (2015) and include indicators designed to measure progress towards achieving climate goals, such as energy efficiency and decarbonization strategies as well as quantifying emissions from industry, transportation, and agriculture.

Emission inventories are constructed by use of economic activity factors (e.g., number of gas wells, power plants, cows) combined with emission factors (emissions per unit emitter). This kind of approach is known as a “bottom-up” approach, and an updated discussion of how inventories are developed is given by Birdsey et al. (2018). Emission estimates are often produced on a country-wide basis and reported to the UNFCCC for use in policy decisions, although some emissions datasets are spatially disaggregated (e.g., EDGAR—Emissions Database for Global Atmospheric Research, Joint Research Center) (Olivier and Janssens-Maenhout 2012; Crippa et al. 2020). Bottom-up emission datasets can have considerable uncertainties, for example, they may not capture all processes leading to emissions. Emissions of CO_2_ occur during combustion of fossil fuels and statistics for fossil fuel consumption are considered relatively, though not perfectly, well known. On the other hand, CH_4_ emissions from fossil fuels result from unintentional leakage or venting during production, storage, and transport and are much more difficult to quantify. New extraction technologies have led to increased natural gas production, focusing attention on leakage from drilling, storage, and transport of fossil fuel (e.g., Alvarez et al. 2012; Peischl et al. 2015; Pétron et al. 2014). In particular, new sources of potential leakage were identified to explain higher emissions inferred from atmospheric observations compared to those estimated using bottom-up methods. Multi-decadal time series of greenhouse gas observations are essential for evaluating emission inventories; however, it is often difficult to use atmospheric concentration to attribute emissions to particular sources. For source attribution, bottom- up inventories are needed and therefore could be considered climate indicators as well. Ideally, bottom-up inventories should be spatially disaggregated so that they can be evaluated for consistency with top-down atmospheric concentration data. Atmospheric observations and emission inventories are complementary for our understanding of the processes and activities affecting climate forcing, and for devising effective and efficient ways to mitigate them (NAS 2018).

### Carbon dioxide

Measurements of air trapped in ice cores and ongoing analysis of modern air samples show that global atmospheric CO_2_ has increased from 280 ppm (parts per million of dry air, MacFarling Meure et al. 2006) in the pre-industrial atmosphere to over 413 ppm as of July 2020 (http://www.esrl.noaa.gov/gmd/ccgg/trends/global.html). This increase has been shown to be due to human activities, especially the burning of fossil fuels with small contributions from cement production (Tans 2009) and land use change. By burning fossil fuels, humans are accelerating the part of the natural geologic carbon cycle that transfers carbon in rocks and sediments to the atmosphere, processes with timescales of ten to hundreds of millennia. Since 1870, cumulative emissions of CO_2_ have totaled 440 ± 20 PgC (Friedlingstein et al. 2019), while cumulative emissions of carbon from land use change since 1750 have been estimated at 205 ± 60 PgC *(*Friedlingstein et al. 2019). Roughly half of anthropogenic emissions remain in the atmosphere with the remainder taken up by the oceans and natural terrestrial biosphere (Ciais et al. 2013). As of 2019, CO_2_ accounts for 2/3 (2.076 Wm^−2^ out of 3.140 Wm^−2^) of the total anthropogenic climate forcing from long-lived gases (not including ozone, aerosols, and clouds) (Hofmann et al. 2006 updated at http://www.esrl.noaa.gov/gmd/aggi/).

### Methane

Current CH_4_ abundance in the atmosphere is unprecedented over at least the last 800,000 years (Loulergue et al. 2008), increasing by 160% since preindustrial times. Atmospheric network observations (mainly NOAA and the AGAGE network) show that global CH_4_ increased rapidly until the late 1990s, leveled off during the early 2000s, and has begun to increase rapidly again since 2006 (Rigby et al. 2008; Dlugokencky et al. 2009). The cause of the renewed atmospheric increase is currently not well understood.

Estimates reported by Saunois et al. (2020) and Jackson et al. (2020) for anthropogenic emissions are 357 TgCH_4_ year^−1^ (range 334–392 TgCH_4_ year^−1^) for 2008–2017. Production of fossil fuels account for 30–35% of all anthropogenic emissions. Livestock, agriculture, and landfills and sewage account for another ~ 60%, with the remainder due to biomass and biofuel burning. A recent study suggested that emissions from fossil fuels and geologic emissions may be 20–60% higher than previously thought, requiring compensating reduction in microbial anthropogenic emissions (Schwietzke et al. 2016) in order to match observed global average CH_4_ abundance and its growth. Natural emissions, mainly from wetlands and terrestrial aquatic systems, are thought to make up ~ 40% of global emissions. CH_4_ has an atmospheric chemical sink that is in approximate balance with its sources, reaction with the hydroxyl radical (OH). All sinks, including small contributions from other reactions and a microbial soil sink, result in about a 10-year atmospheric lifetime of CH_4_. OH has an extremely short atmospheric residence time and is difficult to directly measure. Its global abundance is usually inferred using observations of methyl chloroform (CH_3_CCl_3_), an anthropogenic gas for which emissions are thought to be well known and for which reaction with OH is the main atmospheric loss (e.g., Krol and Lelieveld 2003; Montzka et al. 2011).

CH_4_ contributed 0.52 Wm^−2^ in 2019 to global total anthropogenic radiative forcing, about a quarter of that due to CO_2_ (Hofmann et al. 2006 updated at http://www.esrl.noaa.gov/gmd/aggi/). Including carbon cycle-climate feedbacks, the global warming potential (GWP100) of CH_4_ is 34; on a per mass basis, its emissions are 34 times more effective at trapping heat in the climate system than an equivalent emission of CO_2_ over a 100-year time horizon (Myhre et al. 2013). Due to its atmospheric lifetime of about a decade, CH_4_ has a significantly higher GWP20 (GWP over a 20-year time horizon) of 70. Global temperature change potential (GTP; Shine et al. 2005) is metric comparing the change in surface temperature due to emission pulses of non-CO_2_ GHGs and CO_2_. This metric captures the relative impact of a non-CO_2_ GHG at a particular time usually chosen for policy relevance. The GTP of CH_4_ for 20- and 100-year horizons (including carbon cycle climate feedbacks) is 70 and 11 (Myhre et al. 2013).

### Nitrous oxide

The atmospheric budget of nitrous oxide (N_2_O) is not well understood, and its abundance in the atmosphere is growing at a rate of 0.94 ppb year^−1^ (Dlugokencky, NOAA/GML, www.esrl.noaa.gov/gmd/ccgg/trends_n2o). With an atmospheric lifetime of ~120 years (Davidson and Kanter 2014), it is destroyed mainly in the stratosphere by reaction with excited state atomic oxygen and photolysis. Global natural emissions (ocean upwelling and soil emissions) are thought to be 10–12 Tg N_2_O-N year^−1^ (Davidson and Kanter 2014).

Anthropogenic emissions are estimated at 5.2–5.5 TgN_2_O-N year^−1^. About 66% of this is likely due to agriculture, mostly not only from use of industrially produced fertilizer but also from cultivation of nitrogen-fixing crops (Röckmann and Levin 2005; Park et al. 2012). Another 15% of total emissions come from fossil fuel combustion. Biomass burning contributes 11% of global emissions, with remaining emissions coming from other sources such as waste waters. Population growth nearly guarantees that N_2_O will continue to increase in the atmosphere since producing enough food to feed the growing human population will rely on fertilizer use (Hansen et al. 2017).

N_2_O has a GWP100 of 298 and an atmospheric lifetime of ~120 years (Davidson and Kanter 2014). In 2019, N_2_O contributed 0.2 Wm^−2^ to global anthropogenic radiative forcing, about a tenth of that due to CO_2_ (Hofmann et al. 2006, updated at http://www.esrl.noaa.gov/gmd/aggi/). The GWP20 of N_2_O is relatively close to its GWP100, 268. In terms of global temperature change potential, for N_2_O, GTP100 is 297 and GTP20 is 284. Note that in comparison to CH_4_, the variation among these metrics is small, a reflection of the long atmospheric N_2_O lifetime.

### Halocarbons

Halogenated gases have been produced by industry in significant quantities since the mid-twentieth century to meet societal needs for refrigeration, air conditioning, insulation, solvents and fire protection. Measurements of air extracted from snow in Antarctica confirm that most of these halogenated chemicals (chlorofluorocarbons, hydrochlorofluorocarbons, hydrofluorocarbons, halons, and some chlorinated gases) were not present in the atmosphere in appreciable quantities before the mid-twentieth century.

Many industrially produced halogenated gases are resistant to destruction by natural processes. Once these chemicals escape from refrigerators or foams to the atmosphere, they persist for decades to centuries. They are powerful GHGs, with GWP100s that are up to 14,000 times larger than CO_2_. Atmospheric concentrations of halogenated gases are much lower (by 10^6^) than that of CO_2_, so their contribution to climate forcing today is only about 17% as large as that from CO_2_.

The main means by which halocarbons are destroyed and removed from the atmosphere is by photolysis in the stratosphere leading to reactive forms of chlorine and bromine that deplete stratospheric ozone. This realization led to the 1987 Montreal Protocol on Substances that Deplete the Ozone Layer, and phase-out in the global production of these chemicals. Because halocarbons are also strong GHGs, the Montreal Protocol has been the most successful global climate treaty to date (Velders et al. 2007). Future warming from these compounds will depend on the effectiveness of controls on the next generation of substitutes for ozone-depleting compounds, namely the HFCs. The Kigali Amendment to the Montreal Protocol was agreed to in late 2016 to phase down and stabilize HFC use and associated climate warming but has not yet been ratified by all parties.

## The annual greenhouse gas index

The effect of anthropogenic GHGs on the energy budget of the climate system is quantified using the concept of radiative forcing. The change in radiative forcing over time is a useful climate indicator because it is directly related to the surface temperature change.

Radiative forcing is defined as the change in net radiative flux (downward minus upward in Wm^−2^) at the tropopause due to a perturbation from an external driver, after allowing the stratosphere to come to radiative-dynamical equilibrium (Myhre et al. 2013). Radiative forcing is usually defined relative to 1750, the start of the industrial era. A net positive change results in warming of the climate system. External perturbations include atmospheric concentration changes due to anthropogenic emissions of greenhouse gases, changes in aerosols, and changes in albedo due to land use change. Clouds and water vapor are considered internal to the climate system, but changes in atmospheric temperature and relative humidity, possibly driven by changes in atmospheric composition, can affect cloudiness.

Changes in long-lived, well mixed greenhouse gases, such as CO_2_, CH_4_, N_2_O, and halogenated compounds such as CFCs make up the largest and most certain component of radiative forcing, because their concentrations and atmospheric growth rates can be accurately measured in the atmosphere. The effects of anthropogenic emissions on trace gas atmospheric concentrations and radiative forcing is also relatively well understood in comparison to, for example, the radiative forcing contribution from changing cloudiness and associated cloud-climate feedbacks.

An example of a radiative forcing climate indicator is the NOAA Annual Greenhouse Gas Index (AGGI), which uses long-term global observations of important greenhouse gases to calculate changes in radiative forcing relative to 1990 (the baseline year for the Kyoto Protocol). Total radiative forcing from these main greenhouse gases is calculated using IPCC-recommended expressions derived from atmospheric radiative transfer models to convert greenhouse gas concentration increases relative to 1750 to instantaneous radiative forcing (Ramaswamy et al. 2001). Feedbacks due to water vapor and ozone depletion are not considered in this calculation because they are less well quantified. Other spatially heterogeneous, short-lived, climate forcing agents, such as aerosols and tropospheric ozone, have uncertain global magnitudes and also are not considered in the calculation of the AGGI. Changes in radiative forcing before 1978 are derived from atmospheric measurements of CO_2_ started in the late 1950s (Keeling 1958) and from measurements of CO_2_ and other greenhouse gases in air trapped in snow and ice in Antarctica and Greenland (Etheridge et al. 1996; Butler et al. 1999).

The AGGI was introduced in 2006 based on measurements through 2004 (Hofmann et al. 2006) and has been updated annually. For 2019, the AGGI was 1.45, indicating that total direct radiative forcing of anthropogenic changes in long-lived greenhouse gases since 1750 has increased 45% since 1990.

**Fig. 3:**
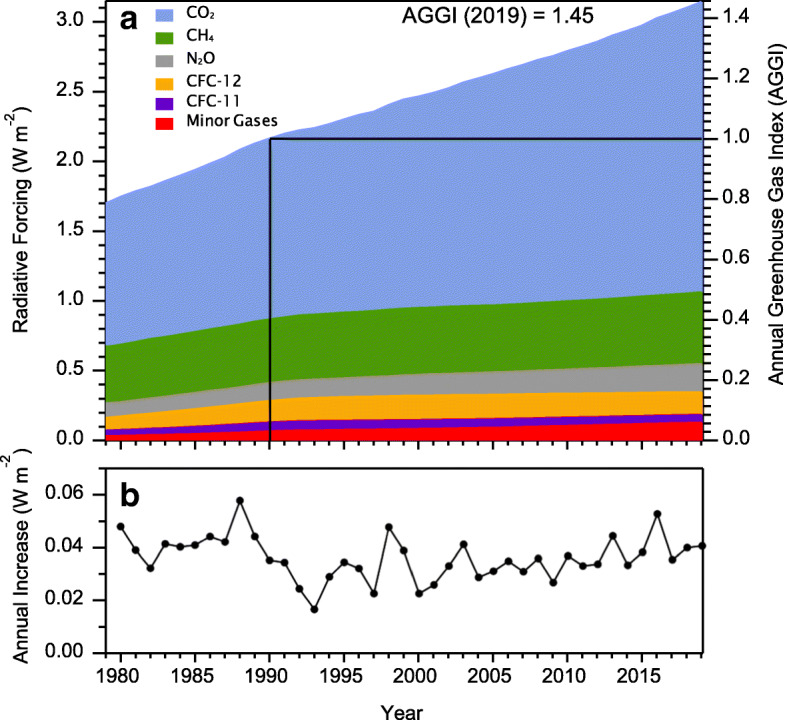
Radiative forcing, relative to 1750, for long-lived greenhouse gases. The NOAA Annual Greenhouse Gas Index (AGGI), which is indexed to 1 for 1990, is shown on the right axis

Figure 3 shows radiative forcing for major GHGs and 15 minor long-lived halogenated gases. Many of these minor gases are also ozone-depleting gases and are regulated by the Montreal Protocol. CO_2_ dominates the total forcing with CH_4_ making up half of the remaining forcing and N_2_O and CFCs accounting for most of the remainder. Five major greenhouse gases (CO_2_, CH_4_, N_2_O, CFC-11, and CFC-12) account for about 96% of the direct radiative forcing by long-lived greenhouse gas increases since 1750. Note that increasing atmospheric CO_2_ accounts for most of the growth in radiative forcing since 1990, about 80%.

Had the ozone-depleting gases not been regulated by the Montreal Protocol and its amendments, it is estimated that climate forcing in 2010 would have been as much as 0.3 W m^−2^ greater in 2010 (Velders et al. 2007), which is more than half of the increase in radiative forcing due to CO_2_ alone since 1990. Of the ozone-depleting gases and their substitutes, the largest contributors to direct warming in 2019 were CFC-12, followed by CFC-11, HCFC-22, CFC-113, and HCFC-134a.

## Potential climate indicators

Cloud climate feedbacks are difficult to quantify, and model predictions are highly uncertain, but understanding cloud-climate feedbacks and effects on the surface radiation budget is critical for quantifying the ultimate effect of greenhouse gases on climate. A potential climate indicator is global dimming/brightening (e.g., Long et al. 2009; Wild 2012, 2009) which quantifies the change in downwelling solar radiation at the Earth’s surface through time, and is an integrated function of all climate forcers and feedbacks, thus providing the highest-level constraint on radiative forcing. Long-term measurements of greenhouse gases, aerosols, and clouds and related climate indicators used with a dimming/brightening index could provide a foundation for quantifying forcing and reducing error in existing indicators that do not account for complicated cloud processes.

Earth system models (ESMs) suggest that there is a near-linear relationship between cumulative emissions of CO_2_ and changes in global surface air temperature (e.g., Allen et al. 2009; Gregory et al. 2009; Matthews et al. 2009). Gillet et al. (2013) showed that this emergent property from the ESMs, referred to as the transient climate response (TCRE), is consistent with both global temperature observations and estimates of cumulative emissions. The TCRE is frequently used to estimate cumulative emission limits for targeted global temperature increases. However, the slopes of the TCRE linear relationships vary among models due to differences in how carbon cycle feedbacks are represented in models (MacDougall et al. 2016; Friedlingstein et al. 2014). Observational constraints on carbon cycle feedbacks could help to constrain TCRE and lead to improvements in ESMs.

About half of all anthropogenic CO_2_ emissions to date have been taken up by the oceans and terrestrial biosphere (Ciais et al. 2013). The size of this “carbon emission discount” will likely change in the future as more carbon accumulates in the oceans, as humans alter landscapes, as climate changes, and atmospheric composition continues to evolve. Efforts to limit global average temperature increases and hopefully avoid the most catastrophic consequences of climate change could be affected by how natural fluxes of carbon and nitrogen change in response to human activities and changing climate.

Coupled carbon cycle–climate models forced with scenarios for future emissions suggest that carbon cycle changes in response to anthropogenic climate change will likely be a positive (additive) feedback on climate, enhancing warming. The magnitude of the projected feedback is uncertain, and a wide range of future atmospheric carbon abundances is predicted by models (Friedlingstein et al. 2014). There are many possible processes that can result in positive carbon cycle feedbacks. A warmer climate can lead to increased fires and droughts, resulting in less uptake and storage of carbon by the terrestrial biosphere. Warming is expected to decrease carbon uptake in the tropics and mid-latitudes (a positive feedback), while in the high latitudes, a warmer climate may lead to increased productivity of the terrestrial biosphere and carbon uptake (a negative feedback), although this may be offset by increases in respiration and release of some of the vast amounts of carbon stored in soils and lakes. Increased atmospheric CO_2_ may cause a fertilization effect on global vegetation leading to increased carbon storage, but the importance of this effect and the factors (e.g., nutrients) which could limit it are highly uncertain.

Land use change can directly affect climate since deforestation and agriculture alter carbon storage in soils and biomass. Fertilizer use also has impacts on the global nitrogen budget and may increase carbon storage while also affecting productivity in marine and freshwater environments as it is washed into marine environments. On the other hand, increased fertilizer use can not only result in increased food production but also increased emissions of the powerful greenhouse gas, N_2_O. Creation or drainage of wetlands can alter CH_4_ emissions (Kolka et al. 2018). For example, drainage of wetlands can lead to lower CH_4_ emissions but higher CO_2_ emissions as a result of lower anerobic respiration and higher aerobic respiration.

Increasing atmospheric CO_2_ also has implications for ocean waters (Cooley et al. 2018). As the partial pressure of CO_2_ in the atmosphere increases, ocean carbon increases and waters become more acidic. Eventually, ocean waters may acidify enough to dissolve calcium carbonate (CaCO_3_) shells of ocean organisms, possibly significantly perturbing ocean ecosystems and reducing productivity of ocean waters.

Frozen Arctic soils are another potentially important carbon cycle-climate feedback (Schuur et al. 2018). An estimated 1700 PgC is frozen in Arctic soils, and warming has proceeded in the Arctic faster than any other region. Current understanding is that ~130–160 PgC, primarily as CO_2_, could be released over the next century (Schuur et al. 2013). On an annual basis, Schuur et al. (2015) estimate that annual carbon releases, if at a constant rate, could be lower than annual fossil fuel emissions (~9 PgCyear^−1^), but comparable to land use change (0.9 PgCyear^−1^). A small percentage of Arctic soil carbon could be released as CH_4_, a more powerful greenhouse gas.

Understanding and identifying feedbacks between GHGs and climate, such as those mentioned above, at a variety of spatial and temporal scales is of first-order importance (Huntzinger et al. 2018). The ability to supply food to a growing global population could be threatened by changes in productivity of marine and land ecosystems. Efforts to reduce GHG emissions could also be made significantly less effective by carbon cycle feedbacks.

Disentangling changes in atmospheric composition that are due to human activities from those due to natural processes is a fundamental scientific challenge. Many GHGs are such as the CFCs, SF_6_, and other halocarbons, are manufactured and do not occur in nature, unlike CO_2_, CH_4_, and N_2_O which have complex sources and sinks that overlap in space and time. GHGs can have removal processes that vary over time, and accounting for these changes is also important when quantitatively estimating the change in radiative forcing due to emissions, especially for short-lived species.

**Fig. 4:**
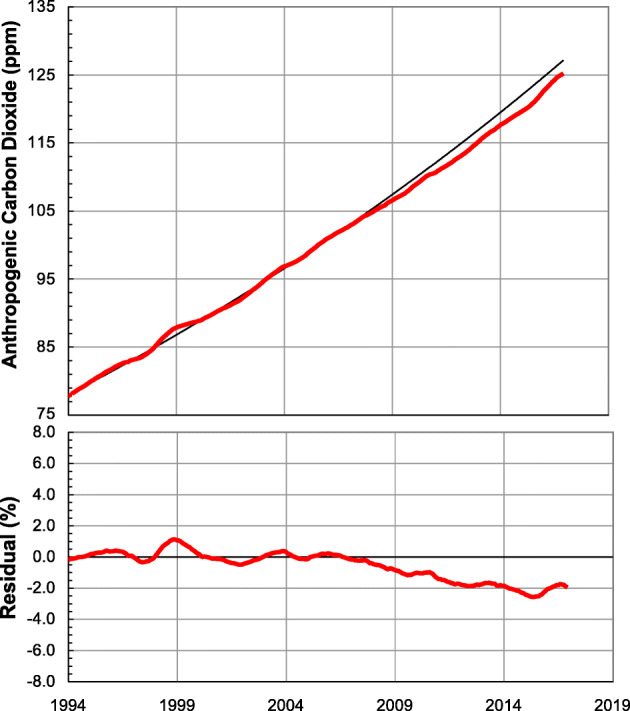
(Top) De-seasonalized global average anthropogenic CO_2_ (red), estimated by subtracting the pre- industrial value of 280 ppm. The black curve shows an exponential fit to observations using the fit parameters of Hofmann et al. (2009) that results in an emission doubling time of about 30 years. (*Bottom*) The difference between the exponential fit of Hofmann et al. (2009) and observed global average CO_2_

A potential climate indicator that could be useful for tracking anthropogenic CO_2_ emissions was introduced by Hofmann et al. (2006), who estimated the anthropogenic contribution to observed global average CO_2_ mole fraction by subtracting the pre-industrial value of 280 ppm. They noted that the resulting anthropogenic CO_2_ mole fraction could be fit to an exponential function with a doubling time of approximately 30 years, and suggested that this doubling time is linked with global population growth. Figure 4 shows an extension of the Hofmann et al. (2009) analysis for recent years using identical fit parameters. A clear trend away from the exponential growth curve can be seen after 2008 implying that growth in anthropogenic CO_2_ is decelerating, possibly due to increased energy efficiency and slower economic growth for these years. Note that in 2015 and 2016, there was an uptick in CO_2_ that is likely related to a strong El Niño that caused a net release of carbon from the terrestrial biosphere as well as economic growth. While this event illustrates the difficulty in separating anthropogenic and natural signals over short time periods as well as further disentangling the response of the land and the ocean components (Chatterjee et al. 2017), longer-term changes are driven almost exclusively by human activities (Tans 2009). It would clearly be ideal to be able to subtract a natural signal from the curves shown in Fig. 4; however, doing so requires either conclusions drawn from uncertain terrestrial and ocean carbon exchange models, or additional observational information.

Observations of trace species with budgets that are related to major GHGs can be helpful for discriminating between anthropogenic and natural contributions. For example, measurements of δ^13^C(CH_4_) can be useful for partitioning emissions between thermogenic (fossil fuels and natural geologic sources) and natural and anthropogenic microbial sources (Schaefer et al. 2016; Nisbet et al. 2016). Radiocarbon (^14^CO_2_ and ^14^CH_4_) can be useful for identifying fossil fuel emissions since all of the ^14^C in fossils fuels has decayed away over the millions of years it took to transform organic to fossil carbon (Graven 2015; Basu et al. 2020). Ethane (C_2_H_6_) and other hydrocarbons can in theory be used to constrain CH_4_ emissions from oil and gas production; however, the emission ratios must be well characterized over space and time (Peischl et al. 2015; Peischl et al. 2016). In addition, COS has been used to constrain uptake of CO_2_ as a result of photosynthesis (Montzka et al. 2007). The use of measurements of related species to constrain GHG budgets is currently an active area of research that may lead to additional robust climate indicators.

### Using remote sensing to monitor atmospheric composition

In situ observational approaches allow for high-quality measurements of atmospheric composition; however, they are limited in spatial and temporal coverage. Over the past decade, remote sensing of atmospheric constituents from satellites has emerged as a potential new technique to quantify the time evolution of atmospheric GHGs. Retrievals of column average abundances from remote sensors use emission or absorption spectra at the top of the atmosphere (TOA). Early GHG satellite instruments such as the Atmospheric Infrared Sounder (AIRS) and the Thermal Emission Sounder (TES) were primarily built for estimating air temperature and water vapor content, and GHG retrievals were incidental benefits from having TOA thermal infrared spectra. The resulting GHG estimates were primarily of the upper troposphere and stratosphere, which are inadequate for estimating surface fluxes.

Satellites such as SCanning Imaging Absorption SpectroMeter for Atmospheric CHartographY (SCIAMACHY), Greenhouse Gases Observing Satellite (GOSAT), TROPOspheric Monitoring Instrument (TROPOMI), and Orbiting Carbon Observatory (OCO) have been built specifically for estimating GHG mole fractions from space. These satellites are sensitive to short wave infrared (SWIR) radiation of sunlight reflected from the Earth’s surface, and provide estimates of the vertical column average CO_2_ and CH_4_ mole fraction including significant sensitivity to the lower troposphere. More such observing systems are planned for the near future, including the Geostationary Carbon Observatory (GeoCARB), MicroCarb, GOSAT-2, and CarbonSat. In theory, GHG estimates from these satellites could be used to track near-surface GHG mole fractions and thereby estimate surface processes behind their fluxes, including their response to climate variability. In practice, the accuracy requirements on satellite sensors of GHGs are daunting, since the signal in the total column that they need to detect is an order of magnitude smaller than the near-surface signal. As a result, small biases (~0.5 ppm for CO_2_ and 1–2 ppb for CH_4_) in total column estimates can significantly alter the inferred surface fluxes of the respective species and skew conclusions about their climate response. Current estimates of CO_2_ and CH_4_ from existing satellite instruments have spatially coherent biases which make it challenging to use them to track carbon climate feedbacks. However, significant progress has been made in this area over the past decade, and it is possible that over the next decade these biases will be further reduced to acceptable levels. Commercial and non-governmental organizations are also developing space-based observing systems with very high spatial resolution (1 km or less) for detecting methane leaks, especially from oil and gas operations (Crisp et al. 2018, App. 5). Examples include GHGSat, MethaneSAT, and microsatellites planned by Bluefield Technologies. Observing systems such as these could provide essential improvements to our ability to quantify fugitive emissions of CH_4_ from anthropogenic sources.

## Conclusions

We propose that long-term observations of atmospheric greenhouse gases be considered as climate indicators, and we have presented a conceptual model showing the relationship between emissions, fluxes, concentration, and radiative forcing. Knowledge of the GHG atmospheric abundances and their change over time allows us to compute and track the associated radiative forcing, as is being done with the Annual Greenhouse Gas Index, for example. GHG abundances and their changes over time also provide valuable information on the success of climate mitigation policies and insights into possible carbon-climate feedback processes that may change the effectiveness of those policies. To ensure that reliable information for assessing GHG emission changes can be provided at need spatial and temporal scales, expanded observational efforts are needed. Furthermore, the ability to detect trends resulting from changing emissions requires a commitment to supporting long-term observations. Efforts to improve and continue bottom-up techniques are also be needed since the interplay of bottom-up and top-down approaches is vital to the success of understanding and mitigating climate change.

## Data Availability

All data used in this study are freely available for download from https://www.esrl.noaa.gov/gmd/.
